# Abnormal regulation of membrane-less organelles contributes to profilin1-associated ALS

**DOI:** 10.1016/j.jbc.2025.110259

**Published:** 2025-05-21

**Authors:** Guoqiang Ma, Xiye Ruan, Bojun Yang, Ningning Li, Dan Su, Shan Sun, Siqian Chen, Kangjia Xu, Zheng Ying, Hongfeng Wang

**Affiliations:** 1 Jiangsu Key Laboratory of Drug Discovery and Translational Research for Brain Diseases, College of Pharmaceutical Sciences, Soochow University, Suzhou, Jiangsu, China; 2 Faculty of Health Sciences, University of Macau, Taipa, Macau, China; 3 MOE Key Laboratory of Geriatric Diseases and Immunology, College of Pharmaceutical Sciences, Suzhou Medical College of Soochow University, Suzhou, Jiangsu, China; 4 Jiangsu Province Engineering Research Center of Precision Diagnostics and Therapeutics Development, Soochow University, Suzhou, Jiangsu, China

**Keywords:** PFN1, amyotrophic lateral sclerosis, membrane-less organelles, stress granule, Cajal body, RNA processing, actin filaments

## Abstract

Profilin 1 (PFN1) is a key cytoskeletal protein that regulates actin dynamics by incorporating monomeric actin into linear filaments. PFN1 deletion or mutations have been linked to numerous neurodegenerative diseases, including amyotrophic lateral sclerosis (ALS). However, the contribution of PFN1 to neurodegenerative pathologies is poorly understood. Recent studies have implicated the role of aberrant cellular membrane-less organelles (MLOs) in neurodegenerative pathogenesis. Here, we demonstrate that PFN1 is involved in the assembly of MLOs, including Cajal bodies and Stress granules. Specifically, depletion of PFN1 leads to abnormal Cajal body accumulation and accelerated maturation into a gel-like state, consequently dysregulating snRNP biogenesis and impairing pre-mRNA splicing efficiency in both neuronal and non-neuronal cells. Similarly, we show that PFN1 knockdown accelerates the assembly of Stress granules in stressed cells. Furthermore, we demonstrate that the ALS-linked PFN1-C71 G mutant exhibits a loss of function in the context of MLO biogenesis. We further reveal that the PFN1 deficiency-induced Cajal body dysregulation, but not Stress granule assembly, is caused by cellular actin filament depolymerization. Importantly, the actin filament agonist CN04 rescues Cajal body properties in PFN1-depleted cells. Taken together, our findings shed light on the role of PFN1 in MLO biogenesis and suggest its involvement in neurodegenerative pathogenesis.

The *PFN1* gene encodes profilin 1 (PFN1), a low-molecular-weight (15 kDa) protein, ubiquitously expressed in mammalian cells (1). PFN1 is involved in various fundamental cellular functions by binding to actin monomers, proline-rich ligands, and phosphoinositides (2, 3, 4, 5). PFN1 was initially identified as a key regulator of actin polymerization and cell migration (6, 7). Recent studies have reported that PFN1 also plays a critical role in other cell processes, including membrane trafficking, cell cycle, proliferation, and autophagy (8, 9). PFN1 deletion or mutations can disrupt normal physiological activities and cause many neurodegenerative diseases, for example, mutations in the PFN1 protein have been linked to the development of amyotrophic lateral sclerosis (ALS) (10, 11, 12). Compared with wild-type, ALS-linked PFN1 variants are highly misfolded and destabilized (13). Meanwhile, in Huntington’s disease (HD), PFN1 maintains huntingtin (Htt) in a soluble state and reduces its aggregation and toxicity by interacting with proline-rich domains (PRD) of Htt (14, 15, 16). Growing evidence indicates that loss of PFN1 is responsible for the progression of HD in patients (17). Moreover, PFN1 expression is reduced in FMRP-depleted mice, a classical disease model related to Fragile-X syndrome (FXS), and overexpression of PFN1 can significantly rescue the developmental defects in this disease model (18, 19, 20).

Dysregulated RNA processing plays a central role in the development of neurodegenerative diseases (21). pre-mRNA splicing is a key step in gene expression, which is mainly catalyzed by the spliceosome, one of the biggest complexes in mammalian cells. The spliceosome contains five small nuclear ribonucleoprotein particles (snRNPs) namely U1, U2, U4, U5, and U6, and other splicing factors. Each snRNP contains one U-rich small nuclear RNA (snRNA) and interacting proteins, like a common set of Sm proteins (B/B′, D1, D2, D3, E, F, and G) (22). The maturation of snRNPs is thought to occur in the Cajal body, a nuclear membrane-less organelle (MLO) (23, 24). ALS-linked *C9ORF72* DPRs have been reported to impair the assembly of Cajal bodies in cells (25, 26).

Compared with the classic membrane-bound organelles (MBOs) such as mitochondria, lysosome, endoplasmic reticulum, and nucleus, MLOs lack a defined membrane boundary and are formed by liquid–liquid phase separation (LLPS) of biomolecular condensates including proteins within low-complexity domains (LCDs) and nucleic acids. MLO disorder has been found in many neurodegenerative diseases (27). For instance, many ALS-related proteins, like TIA1, TAR DNA-binding protein 43 (TDP-43), and Fused in Sarcoma (FUS) are associated with cytoplasmic MLOs, such as Stress granules and P bodies. Disease causal mutations in these genes are shown to alter Stress granule and P body properties and functions, which are relevant to the pathology of ALS. In recent years, we and other groups have reported that *C9ORF72* DPRs cause translocation of the key nucleolar component B23 and NCL, disrupting nucleolar morphology, and inducing nucleolar stress (28, 29, 30). In HD, loss of Stress granules increases mutant Htt aggregation *in vivo* (31). Recent studies also suggested that the Parkinson’s disease protein α-Synuclein directly interacts with P bodies and disrupts mRNA storage and decay in cells (32).

In this study, we show that PFN1 deficiency triggers abnormal Cajal body accumulation and impairs the liquid-like property of Cajal bodies, leading to their dysregulation. Dysregulation of Cajal bodies inhibits snRNP biogenesis and consequently causes pre-mRNA splicing defects. In addition, we find that PFN1 knockdown accelerates Stress granule assembly, demonstrating its role as a negative regulator of Stress granule formation. Moreover, we propose that an ALS-associated PFN1 pathological mutant, PFN1-C71 G, induces the dysregulation of Cajal bodies and triggers Stress granule formation in cells. Furthermore, our study implies a tight connection between actin filament depolymerization and the dysregulation of Cajal bodies caused by PFN1 deficiency. These findings expand the known functions of PFN1 and highlight its novel role in neurodegenerative diseases such as ALS.

## Results

### PFN1 negatively regulates Cajal body formation in non-neuronal and neuronal cells

The PFN1 protein has been found enriched in the nuclear Cajal body in mammalian cells (33). However, it remains unclear whether PFN1 plays a role in Cajal body regulation.

**Figure 1:**
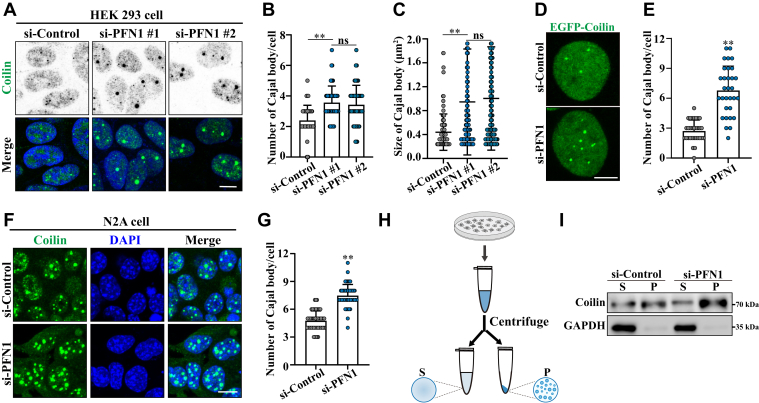
**PFN1 negatively regulates Cajal body formation in non-neuronal and neuronal cells**. *A*, HEK 293 cells were transfected with the indicated siRNAs. After 48 h, the cells were immunostained with an anti-Coilin (Cajal body) antibody. DAPI was used for nuclear staining. The stained cells were visualized by confocal microscopy. Scale bar, 10 μm. *B and C*, quantification of the Cajal body number and size per cell in (*A*). Mean ± SD., ns, not significantly different; ∗∗, *p* < 0.01, *p* values were determined by one-way ANOVA. *D*, HEK 293 cells were transfected with the indicated siRNAs, and expressing EGFP-Coilin. The cells were visualized by confocal microscopy. Scale bar, 10 μm. *E*, quantification of the Cajal body number per cell in (*D*). Mean ± SD., ∗∗, *p* < 0.01, *p* values were determined by unpaired Student’s *t* test. *F*, N2A cells were transfected with the indicated siRNAs. After 48 h, the cells were immunostained with an anti-Coilin (Cajal body) antibody. DAPI was used for nuclear staining. The stained cells were visualized by confocal microscopy. Scale bar, 10 μm. *G*, quantification of the Cajal body number per cell in (*F*). Mean ± SD., ∗∗, *p* < 0.01, *p* values were determined by unpaired Student’s *t* test. *H*, schematic diagram depicting the experimental assay to separate MLOs and supernatant in cells. *I*, HEK 293 cells were transfected with the indicated siRNAs. After 48 h, cell lysates were subjected to droplet co-sedimentation assays and then subjected to immunoblotting using anti-Coilin and GAPDH antibodies (n = 3 biological replicates).

To further investigate whether PFN1 regulates Cajal body formation or maintenance, we used small interfering RNA (siRNA) to knock down the expression of PFN1 in HEK 293 cells. Two siRNA oligos targeting PFN1 were used (si-PFN1#1 and si-PFN1#2), both knocking down PFN1 efficiently (Fig. S1, *A*–*C*). Using an anti-Coilin antibody, the immunofluorescence studies revealed that depletion of PFN1 significantly increased the number and size of Cajal bodies when compared with control cells (Fig. 1, *A*–*C*). Because both PFN1-targeting siRNAs exhibited similar knockdown efficiencies and effects on Cajal body formation, we used si-PFN1#1 (hereafter referred to as si-PFN1) for the remainder of this study. A similar phenomenon was also observed in EGFP-Coilin expressing cells (Fig. 1, *D* and *E*). These data were further confirmed in N2A neuronal cells (Fig. 1, *F* and *G*). Conversely, the specificity of these effects was confirmed by expressing a human siRNA-resistant version of EGFP-PFN1 carrying the NLS signal sequence, which largely impaired Cajal body formation in the nucleus (Fig. S1*D*). Cajal body accumulation upon PFN1 knockdown was confirmed by immunostaining for another commonly used Cajal body marker, TOE1 (Fig. S1, *E* and *F*). Moreover, droplet co-sedimentation analyses confirmed that PFN1 knockdown resulted in more Coilin, as a Cajal body scaffold protein, separated into the dense phase (droplets) from the dilute phase (outside droplets) (Fig. 1, *H* and *I*).

PFN1-depleted cells were also analyzed for changes in other nuclear MLOs, such as Nuclear speckle (SC35), Nucleoli (B23), and PML body (PML). No significant changes in these MLOs were observed in PFN1-depleted cells (Fig. S1, *G*–*M*). Collectively, these data demonstrate that PFN1 negatively regulates Cajal body formation in non-neuronal and neuronal cells.

### PFN1 maintains the liquid-like property of Cajal bodies

**Figure 2:**
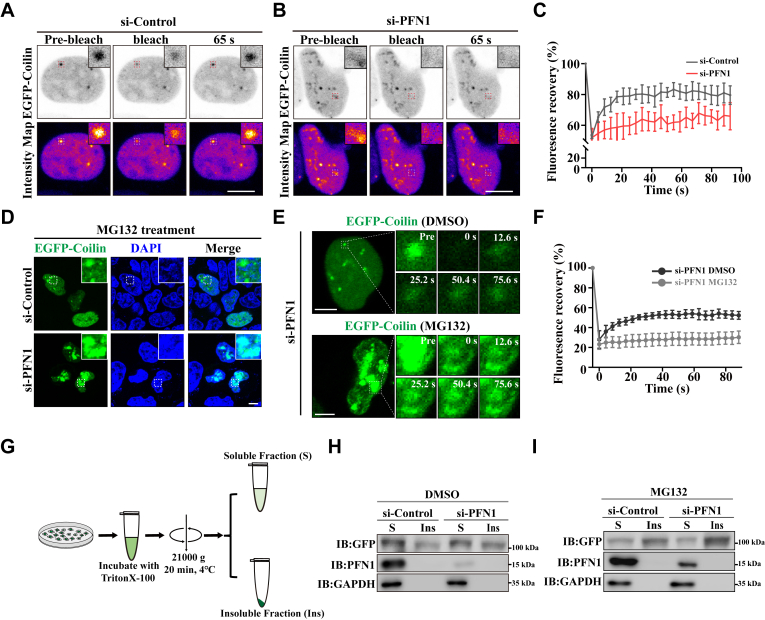
**PFN1 maintains the liquid-like property of Cajal bodies**. *A-B*, FRAP experiments and live cell imaging of EGFP-Coilin in wild-type and PFN1 depleted cells. Scale bar, 10 μm. *C*, FRAP recovery curves (quantification data) of experiments in (*A*-*B*). No statistically significant differences in the size of the Cajal bodies between these two groups. n = 3. *D*, HEK 293 cells were transfected with the indicated siRNAs and expressed EGFP-Coilin. After 48 h, the cells were treated with 10 μM MG132 for 10 h. DAPI was used for nuclear staining. The stained cells were visualized by confocal microscopy. Scale bar, 10 μm. *E*, FRAP experiments and live cell imaging of randomly selected EGFP-Coilin in PFN1 knockdown cells treated with DMSO or MG132. Scale bar, 5 μm. *F*, FRAP recovery curves (quantification data) of experiments in (*E*). n = 4. *G*, schematic diagram depicting the experimental assay to separate TritonX-100 soluble and insoluble protein fractionation in cells. *H and I*, HEK 293 cells were transfected with the indicated siRNAs for 48 h, and then the cells were treated with either DMSO or 10 μM MG132 for another 10 h. Cell lysates were subjected to TritonX-100-soluble and insoluble protein fractionation and subjected to immunoblotting using anti-GFP, PFN1, and GAPDH antibodies (n = 3 biological replicates).

Maintaining the self-assembled proteins in a dynamic liquid-like state is strictly monitored in mammalian cells to support the physiological function of MLOs. Therefore, we used fluorescence recovery after photobleaching (FRAP) and live cell imaging to investigate the potential effects of PFN1 knockdown on the liquid-like property of the accumulated Cajal bodies. FRAP analysis revealed that the fluorescence recovery of EGFP-Coilin droplets was slower in PFN1-depleted cells than in wild-type cells (Fig. 2, *A*–*C*). Furthermore, upon the proteasome inhibitor MG132 treatment, we observed that the irregular Cajal bodies accumulated in PFN1-depleted cells, but less in wild-type cells (Fig. 2*D*). FRAP analysis revealed that the irregular Cajal bodies underwent a transition to solid-like states (Fig. 2, *E* and *F*).

To strengthen our findings, we performed fractionation of cellular proteins into TritonX-100-soluble and insoluble (including protein aggregates) fractions to determine the effects of PFN1 on Cajal body aggregation. We observed that the level of Coilin in the TritonX-100 insoluble fractions was increased in PFN1-depleted cells with or without MG132 treatment (Fig. 2, *G*–*I*). Overall, these results indicate that PFN1 loss disrupts the liquid-like property of Cajal bodies and accelerates Cajal body maturation into a gel even solid-like state.

### PFN1 deficiency causes perturbations in U snRNAs and defects in pre-mRNA splicing

**Figure 3:**
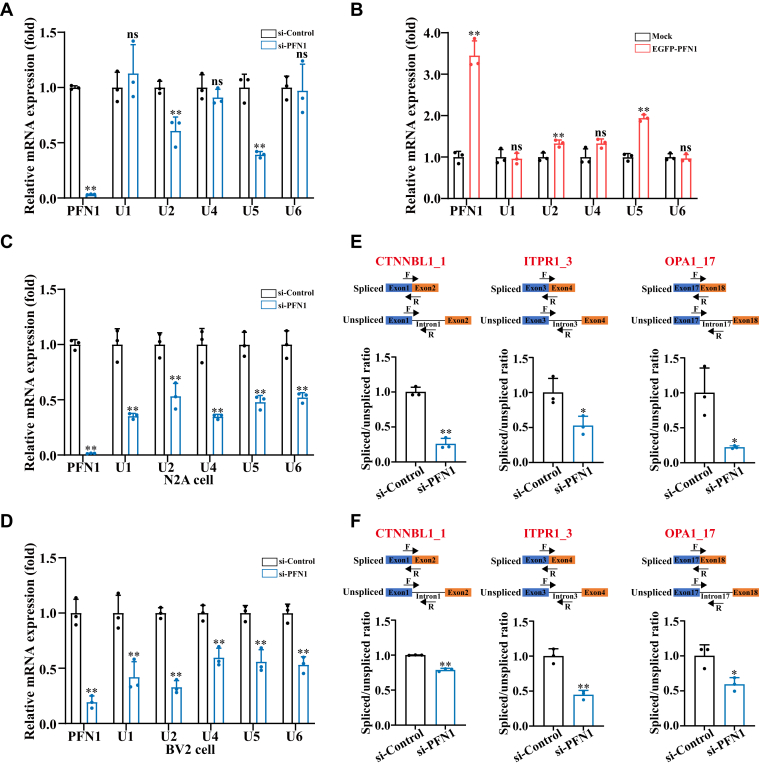
**PFN1 deficiency causes perturbations in U snRNAs and defects in pre-mRNA splicing**. *A*, HEK 293 cells were transfected with the indicated siRNAs. After 48 h, the cells were harvested for qRT–PCR analysis. The data are presented as means ± SD from three independent experiments. ns, not significantly different, ∗∗, *p* < 0.01, *p* values were determined by one-way ANOVA. *B*, HEK 293 cells were transfected with EGFP-PFN1. After 48 h, the cells were harvested for qRT–PCR analysis. The data are presented as means ± SD from three independent experiments. ns, not significantly different, ∗∗, *p* < 0.01, *p* values were determined by one-way ANOVA. *C and D*, N2A cells and BV2 cells were transfected with the indicated siRNAs. After 48 h, the cells were harvested for qRT–PCR analysis. The data are presented as means ± SD from three independent experiments. ∗∗, *p* < 0.01, *p* values were determined by one-way ANOVA. *E and F*, to evaluate the ratio of spliced *versus* unspliced mRNA of the indicated gene, N2A cells and BV2 cells were transfected with the indicated siRNAs. After 48 h, the cells were harvested for qRT-PCR analysis. The data are presented as means ± SD from three independent experiments. ∗, *p* < 0.05, ∗∗, *p* < 0.01, *p* values were determined by unpaired Student’s *t* test.

PFN1 deficiency may alter the liquid property of Cajal bodies, thereby affecting pre-mRNA splicing. The major splicing machinery is U2 type spliceosome, which comprises 5 snRNPs (U1, U2, U4, U5, and U6) (22). To test our hypothesis, we compared the five snRNA levels in PFN1-depleted cells with wild-type cells. Quantitative real-time PCR (qRT–PCR) measurement revealed that U2 and U5 snRNA levels were significantly decreased, and other snRNAs appeared lower but the difference did not reach statistical significance in PFN1-depleted cells (Fig. 3*A*). By contrast, when we overexpressed EGFP-PFN1 in HEK 293 cells, U2 and U5 snRNAs were significantly increased (Fig. 3*B*). Especially, an obvious reduction in the total U snRNA levels was observed in the neuronal cell lines deficient in PFN1 (Fig. 3, *C* and *D*). Moreover, we analyzed the splicing efficiency of three U2-dependent introns in PFN1-depleted N2A and BV2 neuronal cell lines compared with the wild-type cells. The Splicing efficiency was assessed as the ratio of spliced to unspliced pre-mRNA. As we hypothesized, the splicing efficiencies of all three selected U2-dependent introns were substantially reduced by PFN1 knockdown both in N2A and BV2 neuronal cell lines (Fig. 3, *E* and *F*). These results suggest that PFN1 deficiency disrupts U snRNA levels, consequently impairing the efficiency of pre-mRNA splicing.

### PFN1 deficiency accelerates stress granule assembly in response to stress

**Figure 4:**
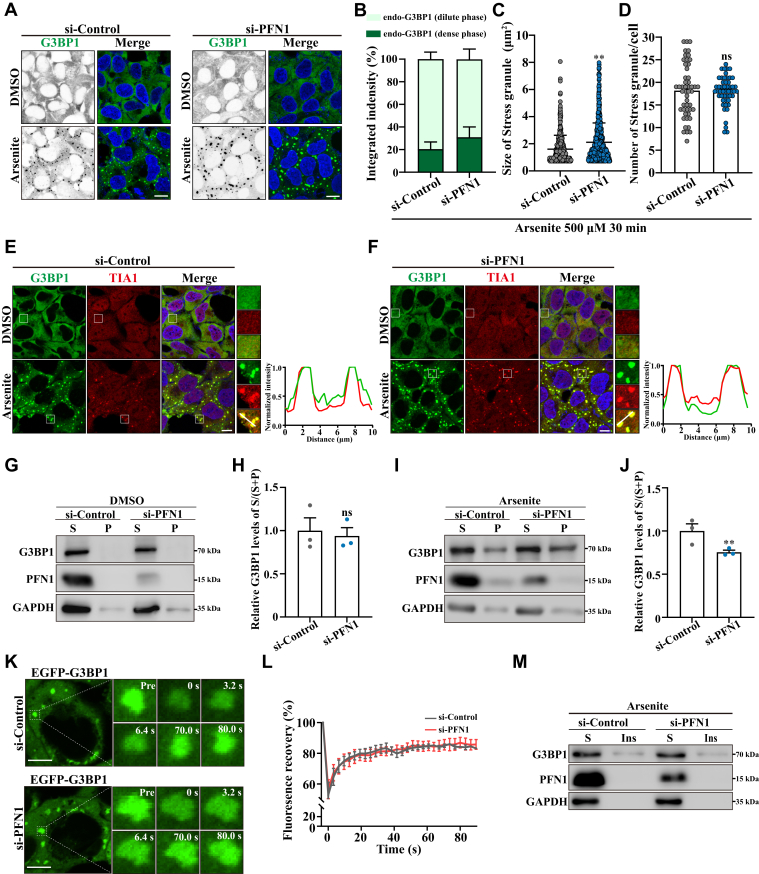
**PFN1 deficiency accelerates Stress granule assembly in response to stress**. *A*, HEK 293 cells were transfected with the indicated siRNAs, and stressed with arsenite (500 μM, 30 min). Subsequently, the cells were fixed, and subjected to immunofluorescence staining using an antibody against G3BP1 (Stress granule), DAPI was used for nuclear staining. The stained cells were visualized by confocal microscopy. Scale bar, 10 μm. *B*, quantification of the integrated fluorescence intensity of G3BP1 in the dense phase (condensates) and dilute phase (surrounding nucleoplasm) of cells in (A). *C*, quantification of the Stress granule size in (*A*). Mean ± SD., ∗∗, *p* < 0.01, *p* values were determined by unpaired Student’s *t* test. *D*, quantification of the Stress granule number in (*A*). Mean ± SD., ns, not significantly different, *p* values were determined by unpaired Student’s *t* test. *E and F*, HEK 293 cells were transfected with the indicated siRNAs and stressed with arsenite (500 μM, 30 min). Subsequently, the cells were fixed and subjected to immunofluorescence staining using antibodies against G3BP1 and TIA1(Stress granule), DAPI was used for nuclear staining. The stained cells were visualized by confocal microscopy. Scale bar, 5 μm. The fluorescence intensities were quantified on the corresponding lines. *G-J*, HEK 293 cells were transfected with the indicated siRNAs for 48 h, and then the cells were treated with either DMSO or 500 μM arsenite for 30 min. Cell lysates were subjected to droplet co-sedimentation assays and then subjected to immunoblotting using anti-G3BP1, PFN1, and GAPDH antibodies. The data are presented as means ± SD from three independent biological replicates. ns, not significantly different, ∗∗, *p* < 0.01, *p* values were determined by unpaired Student’s *t* test. *K*, HEK 293 cells stably expressing EGFP-G3BP1 were transfected with indicated siRNAs. After 48 h, cells were treated with 500 μM arsenite for 30 min to induce Stress granules, and then subjected to FRAP analysis. Scale bars: 5 μm. *L*, FRAP recovery curves (quantification data) of experiments in (*K*). n = 3. *M*, HEK 293 cells were transfected with the indicated siRNAs for 48 h, and then the cells were treated with 500 μM arsenite for 30 min. Cell lysates were subjected to TritonX-100-soluble and insoluble protein fractionation and subjected to immunoblotting using anti-G3BP1, PFN1, and GAPDH antibodies (n = 3 biological replicates).

In mammalian cells, stress granule assembly is a critical cellular process to regulate mRNA stability under stress conditions. Previous reports show that PFN1 is localized in Stress granules following heat shock in U2OS cells (34). However, the relationship between PFN1 and Stress granules is poorly understood. To better define the role of PFN1 in controlling Stress granule formation, we treated cells with arsenite and assessed the kinetics of Stress granule formation *via* immunofluorescence staining of G3BP1 and TIA1 in wild-type cells and PFN1-depleted cells. We observed that cells depleting PFN1 strongly increased the arsenite-induced Stress granule assembly (Fig. 4, *A*–*F*). Control siRNA-treated cells are visibly different compared with the large Stress granules formed in the PFN1-depleted cells following the same stress stimulus, including the different concentrations or times of arsenite treatment (Fig. S2). The same results were also obtained using other types of stress conditions such as proteasome inhibitor (MG132), translation inhibitor (puromycin), exogenous dsRNA stress (Poly(I:C)), or energy depletion (CCCP) (Fig. S3). Moreover, droplet co-sedimentation analyses confirmed that PFN1 knockdown resulted in more G3BP1 separated into the dense phase (droplets) from the dilute phase (outside droplets) (Fig. 4, *G*–*J*). Stress granules display liquid properties similar to other MLOs. To further elucidate the liquid properties of large Stress granules triggered by PFN1 depletion, we utilized GFP-tagged G3BP1 stably expressing cells and employed the FRAP experiment to investigate the mobility of Stress granules during arsenite treatment. The FRAP analysis revealed that GFP-G3BP1 within Stress granules displayed rapid dynamics in both wild-type and PFN1-depleted cells (Fig. 4, *K* and *L*). Furthermore, soluble and insoluble protein fractionation analysis demonstrated that PFN1 knockdown did not disrupt the dynamic nature of Stress granules (Fig. 4*M*). The Stress granule life cycle contains assembly and disassembly, and we demonstrated that PFN1 depletion did not affect Stress granule recovery following arsenite washout (Fig. S4). In conclusion, these data indicate that PFN1 negatively regulates Stress granule assembly upon stress.

The first step in the formation of Stress granules is the phosphorylation of eIF2α at serine 51 by stress-responsive kinases, such as PKR or PERK, resulting in global translation repression and Stress granule formation (35). We, therefore, performed experiments to test whether silencing PFN1 influences eIF2α phosphorylation in response to arsenite treatment. Western blot analysis revealed similar levels of phospho-eIF2α in both wild-type and PFN1-depleted cells upon arsenite treatment (Fig. S5). Thus, the effect of PFN1 on Stress granule assembly is downstream from eIF2α phosphorylation.

### PFN1 modulates Cajal body assembly by regulating actin filament formation

**Figure 5:**
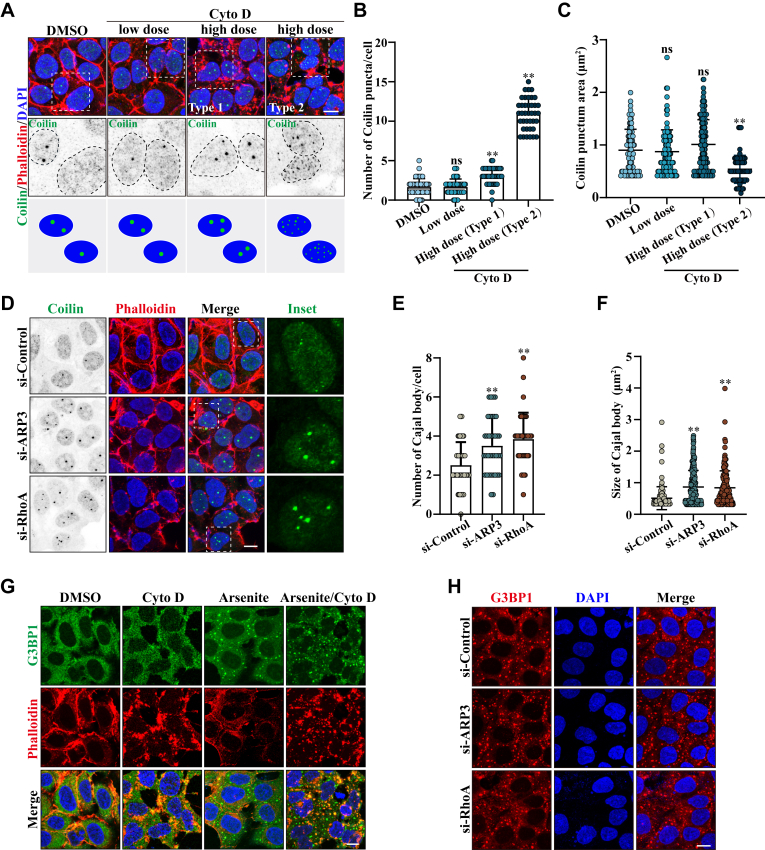
**PFN1 modulates Cajal body assembly by regulating actin filament formation**. *A*, HEK 293 cells were treated with 0.1 μg/ml (low dose) or 1 μg/ml (high dose) Cyto D for 1 h. Subsequently, the cells were fixed, and subjected to immunofluorescence staining using an antibody against Coilin (Cajal body), actin filaments were stained using Phalloidin-iFluor 647, and DAPI was used for nuclear staining. The stained cells were visualized by confocal microscopy. Scale bar, 10 μm. The cartoons below the figures indicate the respective size and number of Cajal body in cells. *B and C*, quantification of the Cajal body number and size per cell in (*A*). Means ± SD., ns, not significantly different; ∗∗, *p* < 0.01, *p* values were determined by one-way ANOVA. *D*, HEK 293 cells were transfected with the indicated siRNAs. After 48 h, the cells were immunostained with an anti-Coilin (Cajal body) antibody. Actin filaments were stained using Phalloidin-iFluor 647, and DAPI was used for nuclear staining. The stained cells were visualized by confocal microscopy. Scale bar, 10 μm. *E and F*, quantification of the Cajal body number and size per cell in (*D*). Means ± SD., ∗∗, *p* < 0.01, *p* values were determined by one-way ANOVA. *G*, HEK 293 cells were treated with 1 μg/ml Cyto D for 1 h and then incubated with 500 μM arsenite for 30 min. Subsequently, the cells were fixed and subjected to immunofluorescence staining using an antibody against G3BP1(Stress granule), actin filaments were stained using Phalloidin-iFluor 647, and DAPI was used for nuclear staining. The stained cells were visualized by confocal microscopy. Scale bar, 10 μm. *H*, HEK 293 cells were transfected with the indicated siRNAs. After 48 h, the cells were treated with 500 μm arsenite for 30 min, then the cells were immunostained with an anti-G3BP1 (Stress granule) antibody. DAPI was used for nuclear staining. The stained cells were visualized by confocal microscopy. Scale bar, 10 μm.

In mammalian cells, PFN1 plays a key role in actin polymerization (36). To investigate whether PFN1 negatively regulates the assembly of Cajal bodies by influencing actin filament assembly, we incubated HEK 293 cells with different doses (0.1, 1 μM) of cytochalasin D (Cyto D), a potent actin polymerization inhibitor, before quantifying Cajal body assembly. We observed that the morphology of the Cajal body remained unchanged following a low dose of Cyto D (0.1 μM) treatment. Nevertheless, we showed that a high dose of Cyto D (1 μM) treatment led to an increase in Cajal bodies in nearly 40% of the cells (Type 1). Additionally, compared to the control cells, 20% to 30% of the cells displayed numerous small Coilin-positive granules (Type 2) (Fig. 5, *A*–*C*). It suggests that PFN1 may modulate Cajal body formation by regulating actin filament formation. To further verify our hypothesis, we knocked down two other actin filament-related genes, ARP3 and RhoA, which have been reported to cooperate with PFN1 to create linear actin filaments (37, 38, 39). The knockdown efficiency of ARP3 or RhoA by si-RNAs was assessed *via* qRT-PCR (Fig. S6). As we assumed, compared with wild-type cells, depletion of ARP3 or RhoA in HEK 293 cells significantly increased the number of Cajal bodies in the nucleoplasm (Fig. 5, *D*–*F*). Above all, our findings reveal that the absence of PFN1 causes actin depolymerization, which is the reason for the Cajal body accumulation.

To determine whether actin depolymerization also contributes to Stress granule dysregulation observed upon PFN1 knockdown, we examined Stress granule morphology following Cyto D treatment or ARP3/RhoA silencing. Neither treatment significantly altered Stress granule morphology induced by arsenite (Fig. 5, *G* and *H*). Therefore, PFN1 deficiency-induced Stress granule dysregulation is independent of actin filaments.

### ALS-linked PFN1 C71 G mutant impairs the assembly and function of cellular MLOs

**Figure 6:**
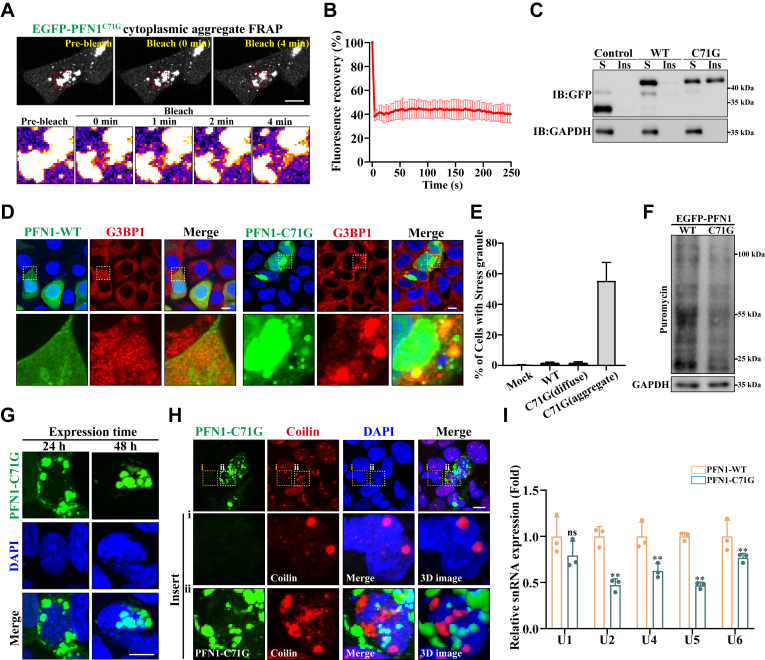
**ALS-linked PFN1-C71 G mutant impairs the assembly and function of cellular MLOs**. *A*, FRAP experiments and live cell imaging of EGFP-PFN1^C71G^ cytoplasmic puncta, Scale bar, 10 μm. *B*, FRAP recovery curves (quantification data) of experiments in (*A*). n = 3. *C*, HEK 293 cells were transfected with EGFP-alone, EGFP-PFN1^WT^, or EGFP-PFN1^C71G^. After 24 h, cell lysates were subjected to soluble and insoluble protein fractionation and immunoblotting using anti-GFP and GAPDH antibodies. *D*, HEK 293 cells were transfected with EGFP-PFN1^WT^ or EGFP-PFN1^C71G^ as indicated. After 24 h, the cells were immunostained with an anti-G3BP1 (Stress granule) antibody. DAPI was used for nuclear staining. The stained cells were visualized by confocal microscopy. Scale bar, 10 μm. *E*, quantification of the percentage of cells with Stress granule in (*D*). *F*, HEK 293 cells expressing EGFP-PFN1^WT^ or EGFP-PFN1^C71G^ were treated with 1 μg/ml puromycin for 15 min, then the cells were subjected to immunoblotting using puromycin and GAPDH antibodies (n = 3 biological replicates). *G*, HEK293 cells were transfected with EGFP-PFN1^C71G^ for the indicated time (24 h or 48 h), then the cells were fixed. DAPI was used for nuclear staining. The stained cells were visualized by confocal microscopy. Scale bar, 10 μm. *H*, HEK293 cells were transfected with EGFP-PFN1^C71G^. After 48 h, the cells were immunostained with an anti-Coilin (Cajal body) antibody. DAPI was used for nuclear staining. The stained cells were visualized by confocal microscopy. Scale bar, 10 μm. *I*, HEK 293 cells were transfected with EGFP-PFN1^WT^ or EGFP-PFN1^C71G^. After 48 h, the cells were harvested for qRT–PCR analysis. The data are presented as means ± SD from three independent experiments. ns, not significantly different, ∗∗, *p* < 0.01, *p* values were determined by one-way ANOVA.

To examine the impact of ALS-associated pathological mutants on cellular MLOs of PFN1, we expressed EGFP-labeled wild-type or ALS-linked PFN1 mutants (A20 T, C71 G, T109 M, M114 T, E117 G, G118 V, R136 V, and Q139 L) in HEK 293 cells. Consistent with previous findings, ALS-linked mutations in PFN1 formed aggregates in the cytoplasm, while wild-type PFN1 was diffusely distributed mainly in the cytoplasm (Fig. S7, *A* and *B*). Among the eight PFN1 mutants, PFN1-C71 G is more prone to form cytoplasmic aggregates in over 30% of PFN1-C71 G expressed cells (Fig. S7, *A* and *B*). These findings were confirmed in two additional neuronal cell lines including N2A cells and SH-SY5Y cells (Fig. S7*C*). FRAP and protein fractionation experiments also implied that the PFN1-C71 G aggregates existed in a solid state (Fig. 6, *A*–*C*). Furthermore, PFN1-C71 G aggregates, but not PFN1-WT or other ALS-linked PFN1 mutants, induced the formation of G3BP1-labeled Stress granules (Fig. 6, *D* and *E*, Fig. S8). It has been reported that Stress granules containing numerous mRNAs and RNA-binding proteins limit translational initiation in response to cellular stress (40). To test whether protein translation is dysregulated in PFN1-C71 G expressed cells, we used the puromycin labeling assay to measure global protein synthesis as previously described (41). Western blot assay of puromycin-labeled nascent proteins revealed a dramatic decrease in translation activity in PFN1-C71 G expressing cells compared to PFN1-WT expressing cells (Fig. 6*F*).

Interestingly, extended expression (48 h) of PFN1-C71 G resulted in the formation of aggregates in both the cytoplasm and the nucleus (Fig. 6*G*). Importantly, the nuclear aggregates of PFN1-C71 G were observed to sequester Cajal bodies, resulting in an irregular morphology of Cajal bodies (Fig. 6*H*). Conversely, other ALS-associated PFN1 mutants did not influence the physiological assembly of Cajal bodies (Fig. S9). Next, to investigate whether PFN1-C71 G affected cellular snRNP biogenesis, we compared the five U snRNA levels in HEK 293 cells expressing PFN1-C71 G with levels in PFN1-WT expressed cells. qRT–PCR measurement revealed that U2, U4, U5, and U6 snRNA levels were significantly decreased in PFN1-C71 G expressed cells (Fig. 6*I*). Overall, these findings suggest that one potential pathogenic mechanism for PFN1-C71 G mutant to initiate ALS might be the dysregulation of MLOs, including Cajal bodies and Stress granules.

### Actin filament agonist CN04 reduces neuronal toxicity caused by PFN1 deficiency

**Figure 7:**
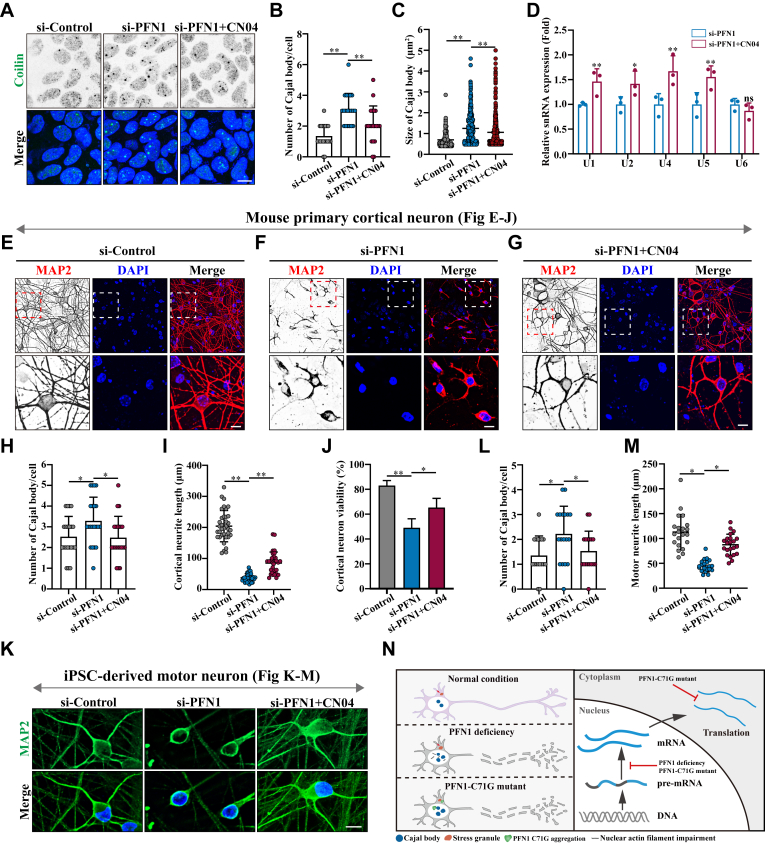
**Actin filament agonist CN04 reduces neuronal toxicity caused by PFN1 deficiency**. *A*, HEK 293 cells were transfected with the indicated siRNAs for 48 h, and then the PFN1-depleted cells were treated with 1 μg/ml CN04 for another 12 h. The cells were immunostained with an anti-Coilin (Cajal body) antibody. DAPI was used for nuclear staining. The stained cells were visualized by confocal microscopy. Scale bar, 10 μm. *B and C*, Quantification of the Cajal body number and size per cell in (*A*). Means ± SD., ∗∗, *p* < 0.01, *p* values were determined by one-way ANOVA. *D*, HEK 293 cells were transfected with the siRNA against PFN1 for 48 h, and then the cells were treated with or without 1 μg/ml CN04 for another 12 h. The cells were harvested for qRT–PCR analysis. The data are presented as means ± SD from three independent experiments. ns, not significantly different, ∗∗, *p* < 0.01, *p* values were determined by one-way ANOVA. *E-G*, mouse primary cortical neurons were transfected with the indicated siRNAs for 48 h, then the neurons were treated with or without 1 μg/ml CN04 for another 12 h. The cells were immunostained with an anti-MAP2 antibody. DAPI was used for nuclear staining. The stained cells were visualized by confocal microscopy. Scale bar, 10 μm. *H*, quantification of the Cajal body number per cell in (*E*-*G*). Means ± SD., ∗, *p* < 0.05, ∗∗, *p* < 0.01, *p* values were determined by one-way ANOVA. (*I*-*J*) Quantification of the neurite length and cell viability in more than 20 neurons (*E*-*G*). Means ± SD., ∗, *p* < 0.05, ∗∗, *p* < 0.01, *p* values were determined by one-way ANOVA. *K*, iPSC-derived motor neurons were transfected with the indicated siRNAs for 48 h, then the neurons were treated with or without 1 μg/ml CN04 for another 12 h. The cells were immunostained with an anti-MAP2 antibody. DAPI was used for nuclear staining. The stained cells were visualized by confocal microscopy. Scale bar, 10 μm. *L and M*, quantification of the number of Cajal bodies and neurite length in more than 20 neurons in (*L*). Means ± SD., ∗, *p* < 0.05, *p* values were determined by one-way ANOVA. *N*, the proposed working model elucidates the role of PFN1 in the context of Cajal body dynamics, Stress granule assembly, and neuronal survival.

CN04, a Rac/Rho A/Cdc42 activator, has been reported to rearrange F-actin filaments in cells (42, 43). To test whether CN04 could rescue the cellular MLO defects (Cajal body and Stress granule) caused by PFN1 deficiency, we treated si-PFN1 cells with or without CN04 and quantified Cajal body and Stress granule assembly. Compared with the control cells, CN04 treatment decreased the number of Cajal bodies in the nucleus (Fig. 7, *A*–*C*). Furthermore, qRT–PCR measurement revealed that CN04 treatment significantly increased the majority of U snRNA levels in the PFN1-depleted cells (Fig. 7*D*). However, CN04 treatment did not notably affect Stress granule assembly in PFN1-depleted cells (Fig. S10).

To further explore the contribution of PFN1 deficiency-medicated Cajal body dysregulation to neurodegeneration, we cultured mouse primary cortical neurons and detected Cajal body formation by immunostaining. We demonstrated that PFN1 depletion increased neuronal Cajal body numbers as in HEK 293 cells (Fig. 7*H*). Moreover, we used MAP2, a classical neuronal marker, to access neurite outgrowth and cell viability in PFN1-deficient neurons. We showed that PFN1-deficient neurons impaired neurite outgrowth and cell viability. Importantly, CN04 treatment significantly restored the property of Cajal bodies and promoted cell viability and neurite outgrowth (Fig. 7, *E*–*J*). In addition, we differentiated the human inducible pluripotent stem cells (iPSCs) into mature motor neurons that express choline acetyltransferase (ChAT), a marker of mature motor neurons (Fig. S11). These findings were further confirmed in iPSC-derived motor neurons (Fig. 7, *K*–*M*). Overall, our results shed light on the significant role of PFN1-medicated cellular MLO quality control in neuronal survival. In the proposed working model: the left panel illustrates that PFN1 deficiency (due to nuclear actin filament impairment) or the C71 G mutation induces a phase transition of Cajal bodies from a liquid to a solid-like state in the nucleus, accelerates Stress granule aggregation in the cytoplasm, and leads to neuronal toxicity. The right panel illustrates that PFN1 deficiency or the C71 G mutation leads to pre-mRNA splicing defects in the nucleus. Additionally, the C71 G mutation inhibits protein translation in the cytoplasm (Fig. 7*N*).

## Discussion

In this study, we propose a previously unknown role for PFN1 in regulating MLO (Cajal bodies and Stress granules) assembly and biological functions. Our data indicate that depletion of PFN1 accelerates Cajal body and Stress granule accumulation, which impairs cell functions and neuronal survival. These results expand the known functions of PFN1 and provide a relationship between aberrant MLOs and the pathogenesis of neurodegenerative diseases.

Neurodegenerative diseases affect the lives of many people worldwide and entail the progressive loss of neuronal cells in the CNS (central nervous system). They are characterized by progressive loss of motor neuron function. Although these diseases affect distinct brain regions and involve the loss of specific neurons and synapses, common features and underlying mechanisms are increasingly recognized (44, 45). Growing evidence indicates that dysregulation of RNA processing is a common pathogenic factor in neurodegenerative diseases (21, 46). For instance, FUS and TDP-43 depletion or abnormal aggregation disrupts mRNA splicing activity and mRNA stability, ultimately leading to neuronal death. Furthermore, the aberrant mRNA splicing event has been identified in HD and FXS patients (41, 47). In mammalian cells, Cajal bodies play an important role in RNA metabolism, especially in RNA splicing (23). Dysregulated Cajal bodies have been observed in numerous neurodegenerative diseases (26, 48, 49). Here, we show that PFN1 is essential for regulating the assembly and function of Cajal bodies. Compared to wild-type cells, depletion of PFN1 causes Cajal body accumulation. PFN1 appears to be specifically required for Cajal body proper assembly, since its depletion does not affect the biogenesis of other nuclear MLOs, such as Nuclear speckles, Nucleoli, and PML bodies (Fig. 1). Furthermore, we demonstrate that the accumulated Cajal bodies lose their liquid properties, undergoing gelation (Fig. 2). The Cajal body has been well established to be involved in the assembly and maturation of splicing machinery, which plays important roles in pre-mRNA splicing (23, 24, 50). A recent study revealed that the dysregulation of BRAT1, a gene linked to neurodegeneration, can disrupt the phase separation and structural integrity of Cajal bodies, thereby impairing snRNP biogenesis and RNA splicing (51). We further demonstrate that the aggregated Cajal bodies negatively affect the formation of snRNPs, consequently disrupting the mRNA splicing process (Fig. 3). We also show that the ALS-linked PFN1-C71 G mutant, but not other ALS-linked PFN1 mutants, forms abnormal nuclear aggregates that disrupt Cajal bodies, thereby impairing snRNP biogenesis (Figure, S9). Along with the Cajal body in the nucleus, the cytoplasm contains various RNP granules involved in RNA metabolism, such as Stress granules (52, 53). Stress granule assembly and functions are related to neurodegenerative diseases, for example, pathological TDP-43 and FUS aggregates show disturbance of Stress granule assembly and biological functions (54). In our research, we demonstrate that TIA1 and G3BP1-positive Stress granules are increased in PFN1-depleted cells (Fig. 4). Meanwhile, PFN1-C71 G forms aggregates in the cytoplasm and triggers Stress granule formation and translational arrest (Figure, S8). In mammalian cells, Cajal bodies and Stress granules are implicated in RNA metabolism, such as RNA splicing and translation (23, 40). Our study suggests that disruption of RNA metabolism could be a potential pathological mechanism underlying neurodegenerative diseases associated with PFN1. In addition to Stress granules, the P body, another type of cytoplasmic MLO, plays a critical role in mRNA metabolism (55). The *ATXN2* gene, characterized by a CAG repeat expansion, is identified as a risk factor associated with ALS. It has been reported that the pathological mutant protein can impair the assembly of P bodies (56). Whether PFN1 also impacts the assembly and biological functions of P bodies warrants further investigation.

Cellular organelle transport and arrangement are mainly achieved through the cytoskeleton (57). In mammalian cells, the endoplasmic reticulum (ER) network rearrangements largely rely on the microtubules, and the morphology of ER could be disrupted using microtubule depolymerizing drug nocodazole (58). Recent studies suggest that MLOs can also use the cytoskeleton for motor-driven transport, and to drive their activity throughout the cells (59). Here, we propose that PFN1 plays a critical role in modulating the assembly of Cajal bodies rather than Stress granules by regulating actin filament formation. These findings shed light on the role of the cytoskeleton in the nuclear MLO organization (Fig. 5). Interestingly, the varied cellular responses of Cajal bodies to high-dose Cyto D treatment reveal the intricate interplay between cytoskeletal dynamics and nuclear MLO organization. Our results suggest the cellular strategies employed in response to cytoskeletal perturbations and warrant further investigation into the regulatory mechanisms connecting actin filaments to nuclear MLO architecture (Fig. 5*A*). Furthermore, we successfully used an actin filament agonist CN04 to restore the Cajal body morphology and function, mitigating neuronal toxicity caused by PFN1 depletion. Our finding that PFN1 regulates MLO assembly through actin filaments provides new insights into its neuroprotective properties (Fig. 7).

Taken together, our findings reveal that PFN1 regulates MLO (Cajal bodies and Stress granules) morphology and biological functions in cells. This not only expands the biological role of PFN1 but also identifies it as a critical factor in neurodegenerative diseases, like ALS, which are characterized by the dysregulation of MLOs.

## Experimental procedures

### Cell culture, transfection, and reagents

Human embryonic kidney 293 (HEK 293) cells, HEK 293 cell line stably expressing EGFP-G3BP1, SH-SY5Y cells, N2A (Neuro-2a) cells, and BV2 cells were cultured in DMEM (Gibco, 11,995,500) supplemented with 10% fetal bovine serum (FBS; allBIO, MN220610) and 1% penicillin-streptomycin at 37 °C in a 5% CO_2_ humidified incubator. All cell lines used in this study were maintained and preserved in our laboratory (60). Hieff Trans Liposomal Transfection Reagent (Yeasen, 40802ES02) was used for plasmid transfection according to the manufacturer's protocol. Cytochalasin D (Cyto D) was purchased from MCE (HY-N6682), MG132 was purchased from Calbiochem (474,790), CN04 was purchased from Cytoskeleton (Denver, CO), puromycin was purchased from MCE (HY-B1743). CCCP (C2759), Poly (I:C) (P1530), and arsenite (S7400) were purchased from Sigma-Aldrich.

### iPSC cell culture and motor neuron differentiation

iPSC Cells were seeded on a 6-well plate coated with Matrigel in the mTeSR1 medium (STEMCELL, 85,850). For iPSC cell passaging, the cells were washed with 1 ml 1x DPBS and added 1 ml 0.02% EDTA at 37 °C for 4 min. Then the cells were washed with 1 ml DMEM/F-12 (Gibco) and cultured in the mTeSR1 medium containing 10 μM ROCK inhibitor Y-27632 (MCE, HY-10071). The mTeSR1 medium was fully changed daily without Y-27632. For the motor neuron differentiation, we used a STEMdiff Motor Neuron Differentiation Kit (STEMCELL, 100–0871) and a STEMdiff Motor Neuron Maturation Kit (STEMCELL, 100–0872) to generate motor neurons according to the manufacturer's protocol.

### Plasmid construction

EGFP-C1-PFN1 was created by subcloning PCR product with BglII/KpnI sites from HA-PFN1 into a pEGFP-C1 vector. Mutants of EGFP-C1-PFN1 (A20 T, C71 G, T109 M, M114 T, E117 G, G118 V, R136 V, Q139 L) were constructed by the site-directed mutagenesis using the ClonExpress MultiS One Step Cloning Kit (Vazyme). EGFP-C1-NLS-PFN1 was created by inserting the NLS sequence (CCAAAAAAGAAGAGAAAGGTA) at the N terminal of MCS in the EGFP-C1-PFN1 plasmid. EGFP-Coilin was generated by excising Coilin cDNA from Flag-Coilin and inserting it into the pEGFP-C1 vector at Xho1/BamH1 sites. Plasmid constructs used in this study were verified by sequencing (GENEWIZ).

### Small-interfering RNA transfection

Gene knockdown experiments by small interfering RNA (siRNA) were carried out by Lipofectamine RNAiMAX (Invitrogen). siRNA sequences were synthesized by Genephrma Company . The siRNA sequences were described as follows: si-Control: 5′-UUCUCCGAACGUGUCACGUTT-3′, si-PFN1 #1 (human): 5′-CCAUAUCCCUCCCGUGUGUTT-3′, si-PFN1 #2 (human) as well as si-PFN1 (mouse): 5′-AGAAGGTGTCCACGGTGGTTT-3′, si-ARP3 (human): 5′-GCCAAAACCUAUUGAUGUATT-3′, si-RhoA (human): 5′-CUAUGAUUAUUAACGAUGUTT-3′.

### Co-sedimentation assay in cells

The cells were harvested and re-suspended with lysis buffer (50 mM Tris-HCl pH 7.6, 150 mM NaCl, 1% NP-40, 1% sodium deoxycholate with the Protease Inhibitor Cocktail (Roche, 4,693,132,001)) on ice, then the lysates were centrifugated at 21,000 g for 20 min at 4 °C and the supernatant (S: Dilute components) were collected. The pellets (P: Dense components) were subsequently washed three times with lysis buffer and collected using lysis buffer.

### Soluble and insoluble protein fractionation

HEK 293 cells were lysed on ice for 1 h with the "Soluble" lysis buffer (10 mM Tris-HCl pH 7.5, 150 mM NaCl, 1% TritonX-100, 10% glycerol), then the lysates were centrifugated at 21,000 g for 20 min at 4°C, the supernatant (S: Soluble fractions) were collected. The pellets (Ins: Insoluble fractions) were subsequently washed twice with "Soluble" lysis buffer and sonicated with "Insoluble" lysis buffer ("Soluble" lysis buffer supplemented with 4% SDS) and collected.

### Translation assay

The global protein synthesis rate was determined by puromycin labeling as previously described (41). In brief, cells were treated with 1 μg/ml puromycin for 15 min, and then the cells were washed twice with ice-cold PBS and lysed in lysis buffer. Puromycin-labeled nascent peptides were detected by immunoblot using an antibody against puromycin.

### Immunoblot

The cells were harvested and lysed on ice with lysis buffer. The protein samples were separated by SDS-PAGE and transferred to polyvinylidene difluoride (PVDF) membranes (Millipore, IPVH00010). The membrane was blocked with 5% fat-free milk in TBST buffer for 30 min and incubated with the primary antibodies overnight at 4 °C. Subsequently, the membrane was incubated with horseradish peroxidase (HRP)-conjugated goat anti-mouse, or anti-rabbit IgG for 2 h. Protein bands were detected by an ECL detection kit (Thermo Fisher, 32,209). The following primary antibodies were used: anti-PFN1 antibody (Proteintech, 67,390-1-lg), anti-Coilin antibody (Proteintech, 10967-1-AP), anti-G3BP1 antibody (Proteintech, 13057-2-AP), anti-GFP antibody (Santa Cruz Biotechnology, sc-9996), anti-eIF2α (Cell Signaling Technology, 9722), anti-Phospho-eIF2α (Cell Signaling Technology, 9721), anti-puromycin antibody (Abclonal, A23031), and anti-GAPDH antibody (Proteintech, 60004-1-Ig). Secondary antibodies were purchased from Jackson Immunoresearch Laboratories, Inc (111–035–062 and 115–035–045). The specificity of each antibody used in our study was validated by the manufacturer.

### Fluorescence recovery after photobleaching (FRAP) and immunofluorescence

FRAP experiments were performed on a Nikon A1 HD25 confocal microscope with × 63 oil immersion objective. For FRAP in living cells, the cells transfected with EGFP-tagged proteins were grown on glass bottom dishes. Then the selected regions were photobleached with a laser intensity of 70% at 488 nm for 10 s and recovery was recorded for the recorded time. An autofocus system was used during the FRAP experiments. FRAP recovery curves were performed using ImageJ software. For immunofluorescence staining, the cells were fixed with 4% paraformaldehyde (PFA) for 10 min at room temperature and permeabilized with 0.1% TritonX-100 for 10 min. Then the cells were incubated with anti-Coilin (Proteintech, 10967-1-AP), anti-TOE1 (Proteintech, 16203-1-AP), anti-SC35 (Sigma, S4045), anti-B23 (Proteintech, 60096-1-Ig), anti-PML (Santa Cruz, sc-377340), anti-G3BP1 (Proteintech, 13057-2-AP), anti-G3BP1 (Santa Cruz, sc-365338), anti-TIA1 (Proteintech, 12133-2-AP), anti-ChAT (Millipore, AB144P), and anti-MAP2 (Proteintech, 67,015-1-lg) overnight at 4 °C. After washing with PBST, cells were incubated with the fluorescence-labeled secondary antibodies for 3 h at room temperature in a dark humid chamber. F-actin was stained with Alexa iFluor 647-phalloidin (Abcam, ab176759) for 20 min. The 4′,6-diamidino-2-phenylindole (DAPI) was used for nucleus staining. Images were performed using a Nikon A1 HD25 confocal microscope. The fluorescence images were processed for 3D reconstruction using Imaris software. The specificity of each antibody used in our study was validated by the manufacturer.

### RNA isolation, reverse transcription, and quantitative PCR

Total RNA was extracted from cells using the Trizol reagent and subjected to reverse transcription (RT) using the PrimeScript RT kit (Vazyme). qRT-PCRs were performed using the SYBR Green PCR reagent mix (Vazyme, China). The primer sequences were described as follow: human PFN1: 5′-GGGTGGAACGCCTACATCG-3′ and 5′-CCATTCACGTAAAAACTTGACCG-3′, human/mouse U1: 5′-GATACCATGATCACGAAGGTGGTT-3′ and 5′-CACAAATTATGCAGTCGAGTTTCC-3′, human/mouse U2: 5′-TTTGGCTAAGATCAAGTGTAGTATCTGTTC-3′ and 5′-AATCCATTTAATATATTGTCCTCGGATAGA-3′, human U4: 5′-GCCAATGAGGTTTATCCGAGG-3′ and 5′-TCAAAAATTGCCAATGCCG-3′, mouse U4: 5′-GCGATTATTGCTAATTGAAA-3′ and 5′-AAAAATTGCCAATGCCGACTA-3′, human U5: 5′-GGTTTCTCTTCAGATCGTATAAATC-3′ and 5′-CTCAAAAAATTGGTTTAAGACTCAGA-3′, mouse U5: 5′-TACTCTGGTTTCTCTTCAGATCGTATAAAT-3′ and 5′-AATTGGTTTAAGACTCAGAGTTGTTCCT-3′, human U6: 5′-CTCGCTTCGGCAGCACA-3′ and 5′-AACGCTTCACGAATTTGCGT-3′, mouse U6: 5′-GCTTCGGCAGCACATATACTAAAAT-3′ and 5′-ACGAATTTGCGTGTCATCCTT-3′, human ARP3: 5′-GGAGAACGGACGTTGACCG-3′ and 5′-TCCTGCGATTGGAATGTGTTT-3′, human RhoA: 5′-AGCCTGTGGAAAGACATGCTT-3′ and 5′-TCAAACACTGTGGGCACATAC-3′, mouse PFN1: 5′-AACGCCTACATCGACAGCC-3′ and 5′-CGTAATGCTAACGAAGGTCTTCC-3′, mouse CTNNBL1 spliced: 5′-ACTTTTAAGCTACCAGCCCA-3′ and 5′-TCTCGAGGACCAGTTTGCTT-3′, mouse CTNNBL1 unspliced: 5′-GAACTTTTAAGCTACCAGGTAA-3′ and 5′-CAAGATGACGGGACTCTGT-3′, mouse ITPR1 spliced: 5′-TTATCAGCACCTTAGGCTTGG-3′ and 5′-TGAATTTCTTGGGTGGATTGTTAA-3′, mouse ITPR1 unspliced: 5′-GATTTATCAGCACCTTAGGGTAAG-3′ and 5′-CTTGGAAAAGCCACTTAATGTG-3′, mouse OPA1 spliced: 5′-CAGAATTCAAAACTGCTAAAGACAA-3′ and 5′-GAACCATTTTCCAAAAGCAGT-3′, mouse OPA1 unspliced: 5′-ATTCAAAACTGCTAAAGTAGGT-3′ and 5′-GATTGATAAAGCGGAAGCAGTT-3′, human GAPDH: 5′-AAATCCCATCACCATCTTCCAG-3′ and 5′-AGGGGCCATCCACAGTCTTCT-3′, mouse GAPDH: 5′-TGTGTCCGTCGTGGATCTGA-3′ and 5′-TTGCTGTTGAAGTCGCAGGAG-3′.

### Statistical analysis

GraphPad Prism software (version 9.0) was used for statistical analysis. Data were analyzed by two-tailed unpaired *t* test or one-way ANOVA, and *p*-values were indicated in figure legends. Error bars represent the standard deviation (SD). The immunoblotting data were quantified using ImageJ software.

## Data availability

All data described have been included in the manuscript.

## Supporting information

This article contains supporting information.

## Conflict of interest

The authors declare that they have no conflicts of interest with the contents of this article.
