# Supplementary material for: Abnormal regulation of membrane-less organelles contributes to profilin1-associated ALS
Source: J Biol Chem. 2025 May 21;301(7):110259. doi: 10.1016/j.jbc.2025.110259 (PMC12221373; doi:10.1016/j.jbc.2025.110259)
Supplement: Supporting Information [file mmc1.pdf]

Supporting Information

Abnormal regulation of membrane-less organelles contributes to profilin1-assoicated ALS

Guoqiang Ma, Xiye Ruan, Bojun Yang, Ningning Li, Dan Su, Shan Sun, Siqian Chen, Kangjia Xu, Zheng Ying *, Hongfeng Wang *

Figure S1

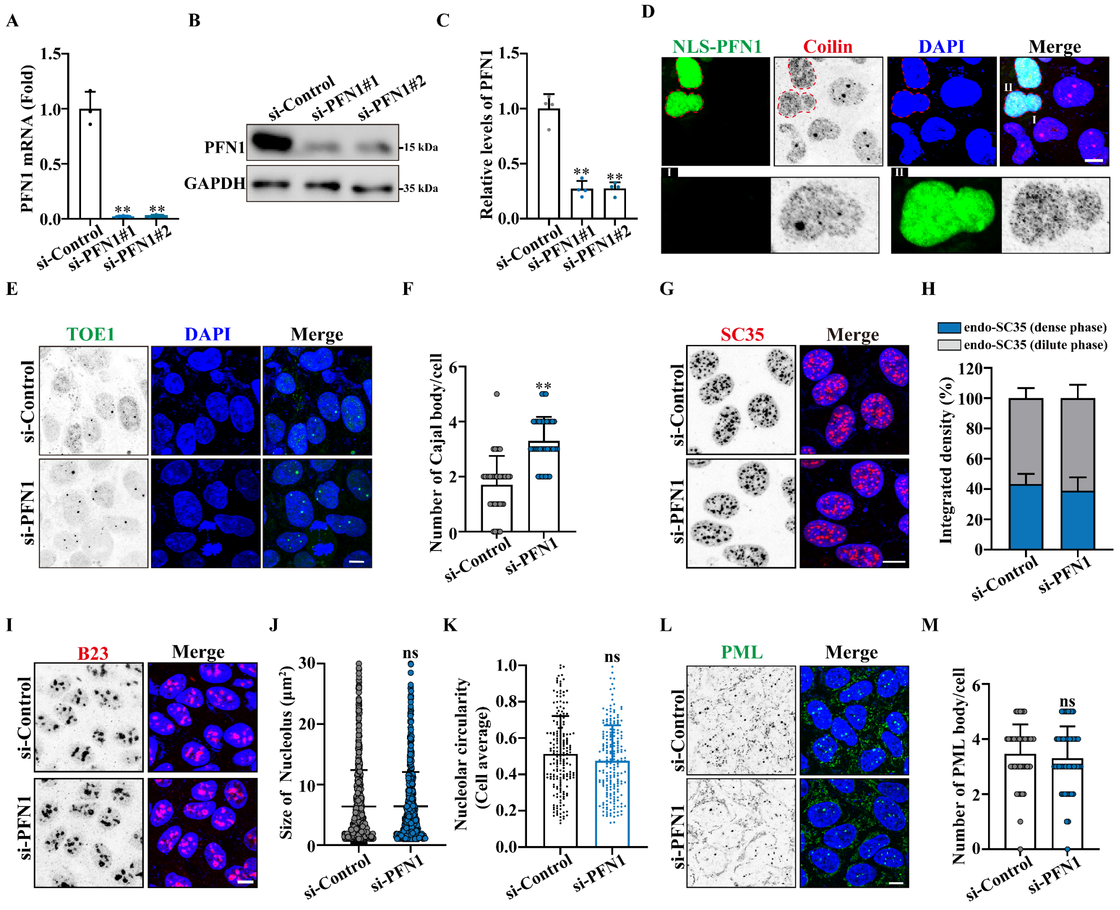

Figure S1. Related to Figure 1. (A) HEK 293 cells were transfected with the indicated siRNAs. After 48 h, the cells were harvested for qRT–PCR analysis. The relative expression level of PFN1 mRNA was quantified by normalizing to GAPDH mRNA. The data are presented as means ± SD from three independent experiments. **, p<0.01, p values were determined by one-way ANOVA. **(B)** HEK 293 cells were transfected with the indicated siRNAs. After 48 h, the cells were subjected to immunoblotting with PFN1 and GAPDH antibodies. **(C)** Quantitative analysis of PFN1 protein level in (B). The data are presented as means ± SD from four independent experiments. **, p<0.01, p values were determined by one-way ANOVA. **(D)** HEK 293 cells were transfected with the indicated siRNAs, and re-transfected with EGFP-C1-NLS-PFN1. After 48 h, the cells were immunostained with an anti-Coilin (Cajal body) antibody. DAPI was used for nuclear staining. The stained cells were visualized by confocal microscopy. Scale bar, 10 μm. **(E)** HEK 293 cells were transfected with the indicated siRNAs. After 48 h, the cells were immunostained with an anti-TOE1 (Cajal body) antibody. DAPI was used for nuclear staining. The stained cells were visualized by confocal microscopy. Scale bar, 10 μm. **(F)** Quantification of the Cajal body number per cell in (E). Means ± SD, **, p<0.01, p values were determined by unpaired Student's *t* test. **(G)** HEK 293 cells were transfected with the indicated siRNAs. After 48 h, the cells were immunostained with an anti-SC35 (Nuclear speckle) antibody. DAPI was used for nuclear staining. The stained cells were visualized by confocal microscopy. Scale bar, 10 μm. **(H)** Quantification of the integrated fluorescence

intensity of SC35 in the dense phase (condensates) and dilute phase (surrounding nucleoplasm) of cells in (G). **(I)** HEK 293 cells were transfected with the indicated siRNAs. After 48 h, the cells were immunostained with an anti-B23 (Nucleus) antibody. DAPI was used for nuclear staining. The stained cells were visualized by confocal microscopy. Scale bar, 10 μm. **(J)** Quantification of the nucleolus size per cell in (I). Means ± SD., ns, not significantly different, p values were determined by unpaired Student's *t* test . **(K)** Quantification of the nucleolar circularity per cell in (I). Means ± SD., ns, not significantly different, p values were determined by unpaired Student's *t* test. **(L)** HEK 293 cells were transfected with the indicated siRNAs. After 48 h, the cells were immunostained with an anti-PML (PML Body) antibody. DAPI was used for nuclear staining. The stained cells were visualized by confocal microscopy. Scale bar, 10 μm. **(M)** Quantification of the PML Body number and size per cell in (L). Means ± SD., ns, not significantly different, p values were determined by unpaired Student's *t* test.

Figure S2

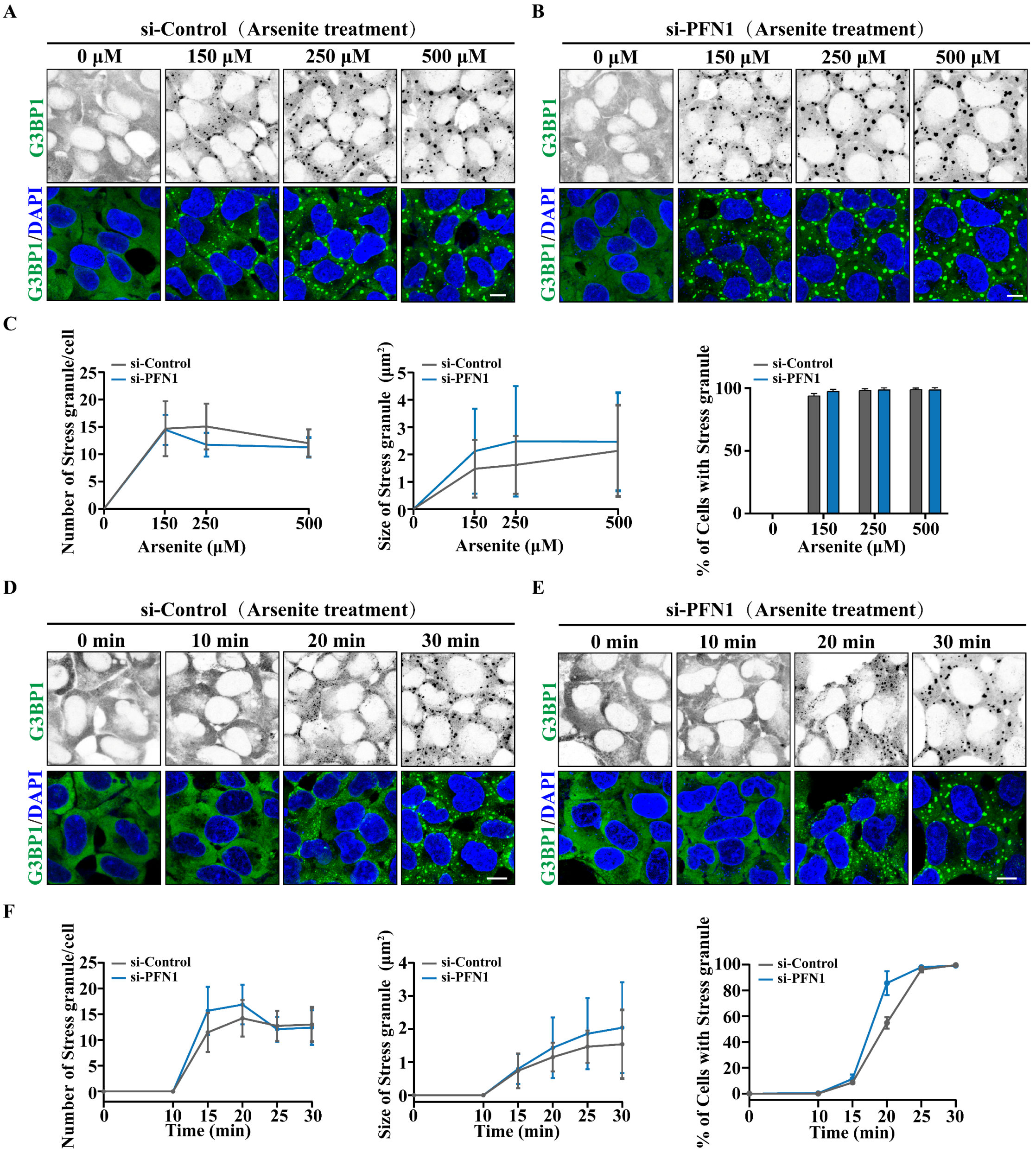

Figure S2. Related to Figure 4. (A and B) HEK 293 cells were transfected with the indicated siRNAs and stressed with indicated concentrations of arsenite for 30 min. Subsequently, the cells were fixed, and subjected to immunofluorescence staining using an antibody against G3BP1(Stress granule), DAPI was used for nuclear staining. The stained cells were visualized by confocal microscopy. Scale bar, 10 μm. **(C)** Quantification of the Stress granule number, size, and percentage of cells with Stress granule in (A and B). Mean ± SD. **(D and E)** HEK293 cells were transfected with the indicated siRNAs and stressed with 500 μM arsenite for indicated times and stained for G3BP1. Subsequently, the cells were fixed, and subjected to immunofluorescence staining using an antibody against G3BP1(Stress granule), DAPI was used for nuclear staining. The stained cells were visualized by confocal microscopy. Scale bar, 10 μm. **(F)** Quantification of the Stress granule number, size, and percentage of cells with Stress granule in (D and E). Mean ± SD.

Figure S3

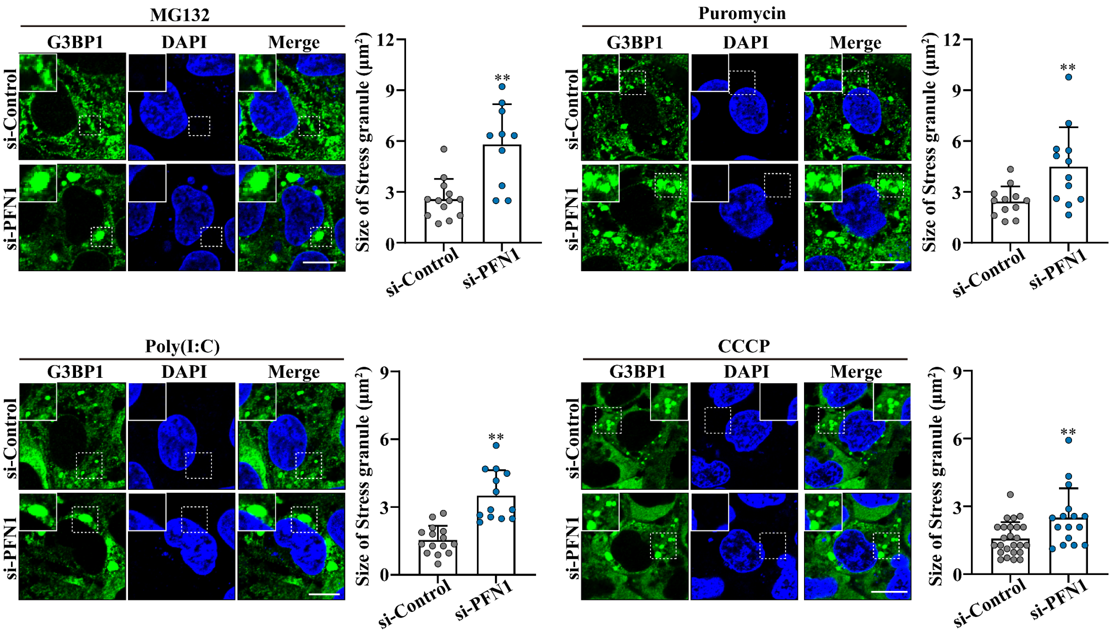

Figure S3. Related to Figure 4. HEK 293 cells were transfected with the indicated siRNAs and stressed with various types of stress, 10 μM MG132 for 3 h, 20 μg/ml puromycin for 3 h, poly(I:C) (2 μg/ml) for 10 h, 20 μM CCCP in glucose-free medium for 1 h. Subsequently, the cells were fixed, and subjected to immunofluorescence staining using an antibody against G3BP1(Stress granule), DAPI was used for nuclear staining. The stained cells were visualized by confocal microscopy. Scale bar, 10 μm. The size of Stress granule in cells were counted. Mean ± SD., **, p<0.01, p values were determined by unpaired Student's *t* test.

Figure S4

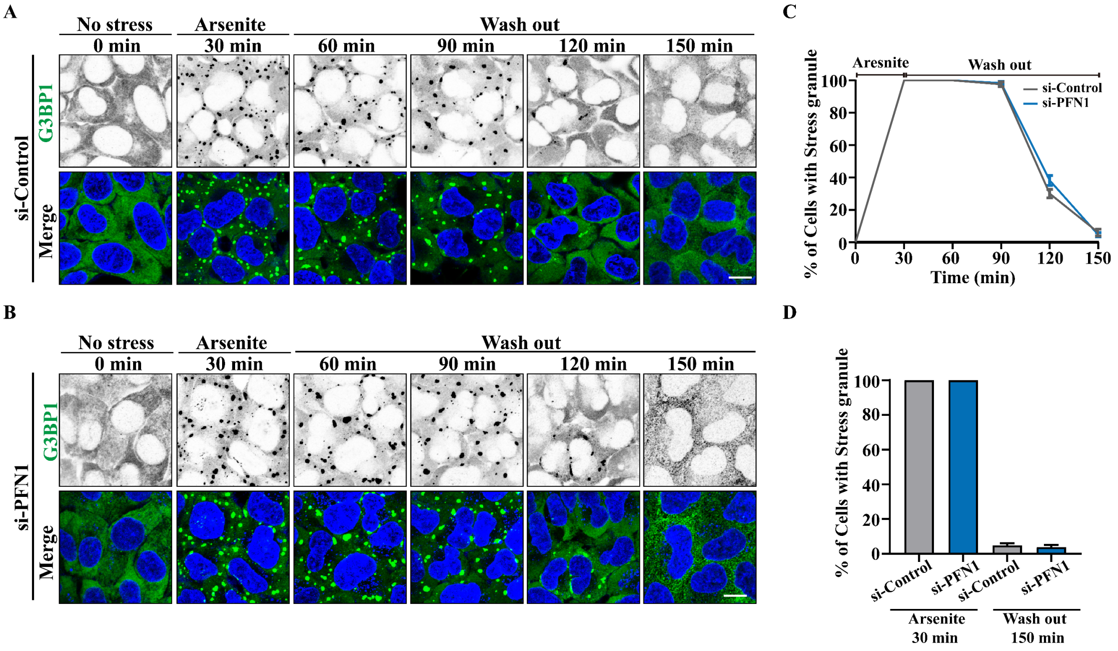

Figure S4. Related to Figure 4. (A and B) HEK 293 cells were transfected with indicated siRNAs. Cells were treated with 500 μM arsenite for 30 min, and the medium was replaced with a normal culture medium to recover for the indicated time, respectively. The cells were fixed, subjected to immunofluorescence staining using an antibody against G3BP1(Stress granule), DAPI was used for nuclear staining. The stained cells were visualized by confocal microscopy. Scale bar, 10 μm. **(C and D)** Quantification of the percentage of cells with Stress granule in (A and B). Mean ± SD.

Figure S5

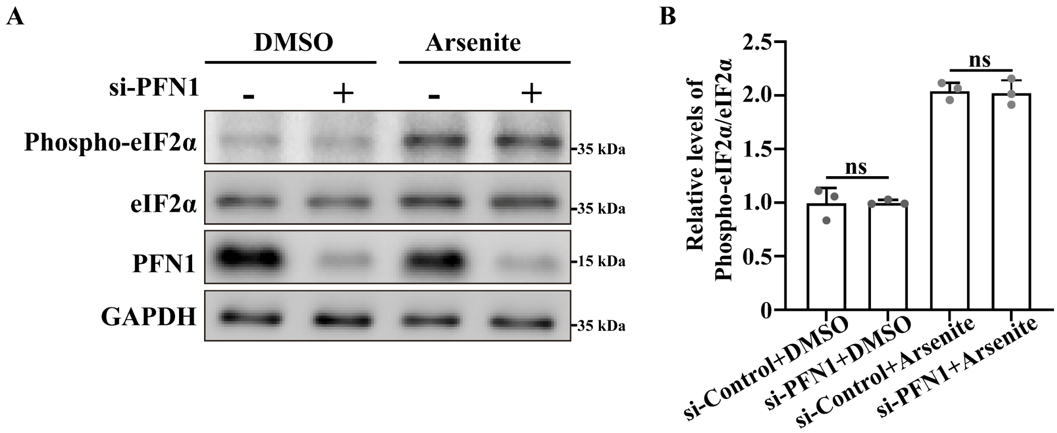

Figure S5. Related to Figure 4. (A and B) HEK 293 cells were transfected with the indicated siRNAs for 48 h, and then the cells were treated with either DMSO or 500 μM arsenite for 30 min. The cells were subjected to immunoblotting using Phospho-eIF2α, eIF2α, PFN1, and GAPDH antibodies. The data are presented as means ± SD from three independent biological replicates. ns, not significantly different, p values were determined by one-way ANOVA.

Figure S6

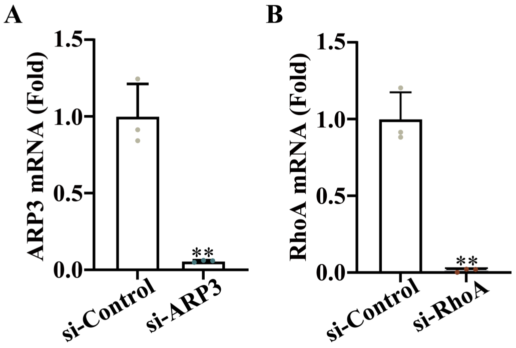

Figure S6. Related to Figure 5. (A and B) HEK 293 cells were transfected with the indicated siRNAs. After 48 h, the cells were harvested for qRT–PCR analysis. The relative expression level of ARP3 or RhoA mRNA was quantified by normalizing to GAPDH mRNA. The data are presented as means ± SD from three independent experiments. **, p<0.01, p values were determined by unpaired Student's *t* test.

Figure S7

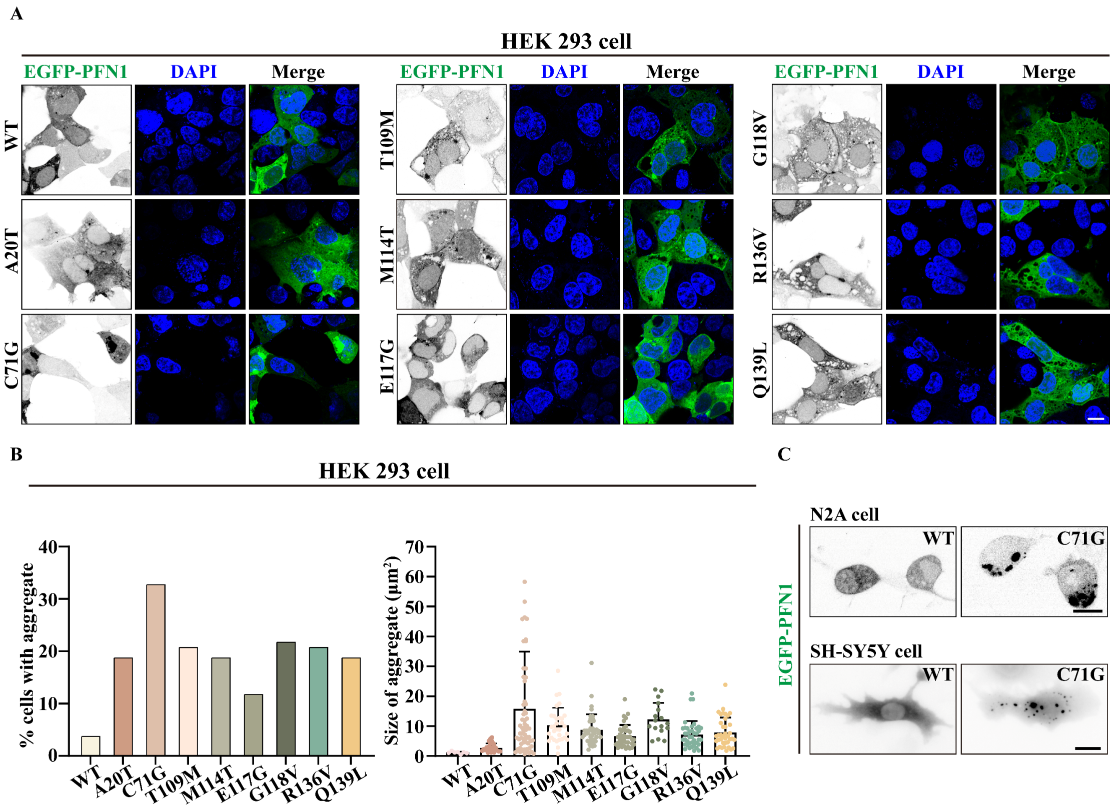

Figure S7. Related to Figure 6. (A) HEK 293 cells were transfected with EGFP-PFN1^{WT} or ALS-linked PFN1 mutants (A20T, C71G, T109M, M114T, E117G, G118V, R136V, Q139L). DAPI was used for nuclear staining. The stained cells were visualized by confocal microscopy. Scale bar, 10 μm. **(B)** Quantification of the cytoplasmic aggregate size and percentage of cells with aggregates in (A). **(C)** N2A cells and SH-SY5Y cells were transfected with EGFP-PFN1^{WT} or EGFP-PFN1^{C71G}, the cells were visualized by confocal microscopy. Scale bar, 10 μm.

Figure S8

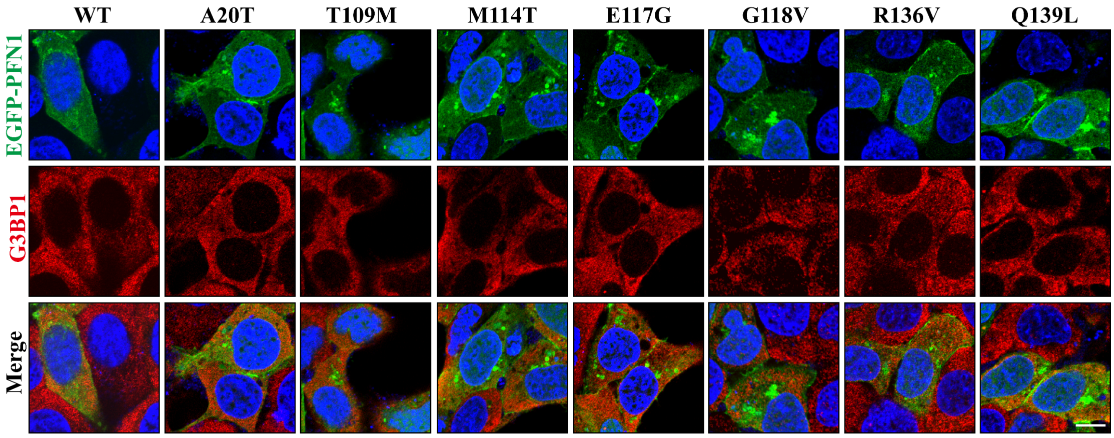

Figure S8. Related to Figure 6. HEK 293 cells were transfected with EGFP-PFN1^{WT} or ALS-linked PFN1 mutants (WT, A20T, T109M, M114T, E117G, G118V, R136V, Q139L). After 24 h, the cells were immunostained with an anti-G3BP1 (Stress granule) antibody. DAPI was used for nuclear staining. The stained cells were visualized by confocal microscopy. Scale bar, 10 μm.

Figure S9

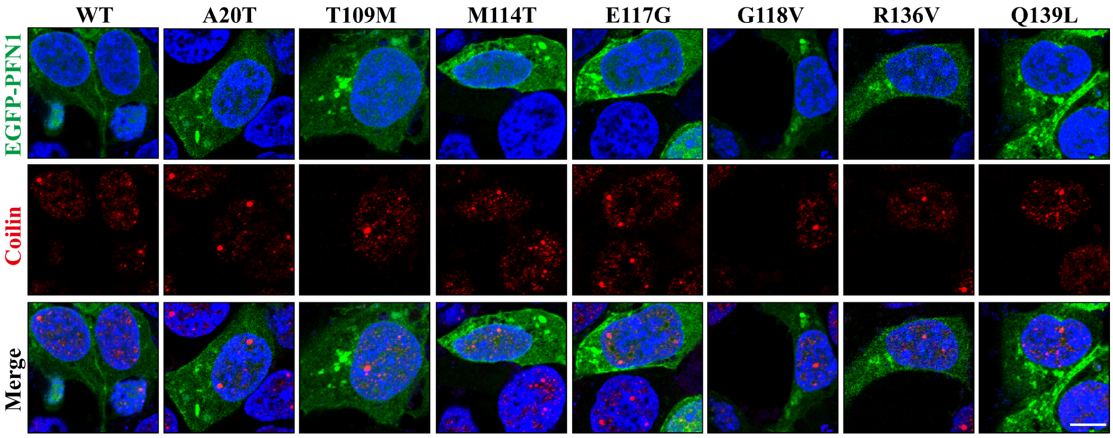

Figure S9. Related to Figure 6. HEK 293 cells were transfected with EGFP-PFN1^{WT} or ALS-linked PFN1 mutants (WT, A20T, T109M, M114T, E117G, G118V, R136V, Q139L). After 48 h, the cells were immunostained with an anti-Coilin (Cajal body) antibody. DAPI was used for nuclear staining. The stained cells were visualized by confocal microscopy. Scale bar, 10 μm.

Figure S10

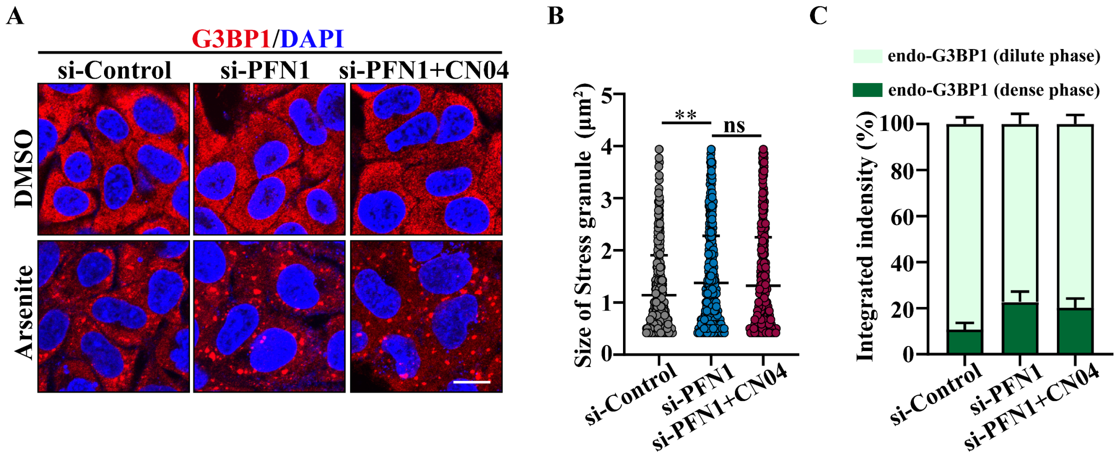

Figure S10. Related to Figure 7. (A) HEK 293 cells were transfected with the indicated siRNAs for 48 h, and then the cells were treated with or without 1 μg/ml CN04 for another 12 h before being incubated with either DMSO or 400 μM arsenite for the last 0.5 h. The cells were immunostained with an anti-G3BP1 (Stress granule) antibody. DAPI was used for nuclear staining. The stained cells were visualized by confocal microscopy. Scale bar, 10 μm. **(B)** Quantification of the Stress granule size in (A). Mean ± SD., ns, not significantly different, **, $p < 0.01$, p values were determined by one-way ANOVA. **(C)** Quantification of the integrated fluorescence intensity of G3BP1 in the dense phase (condensates) and dilute phase (surrounding nucleoplasm) of cells in (A).

Figure S11

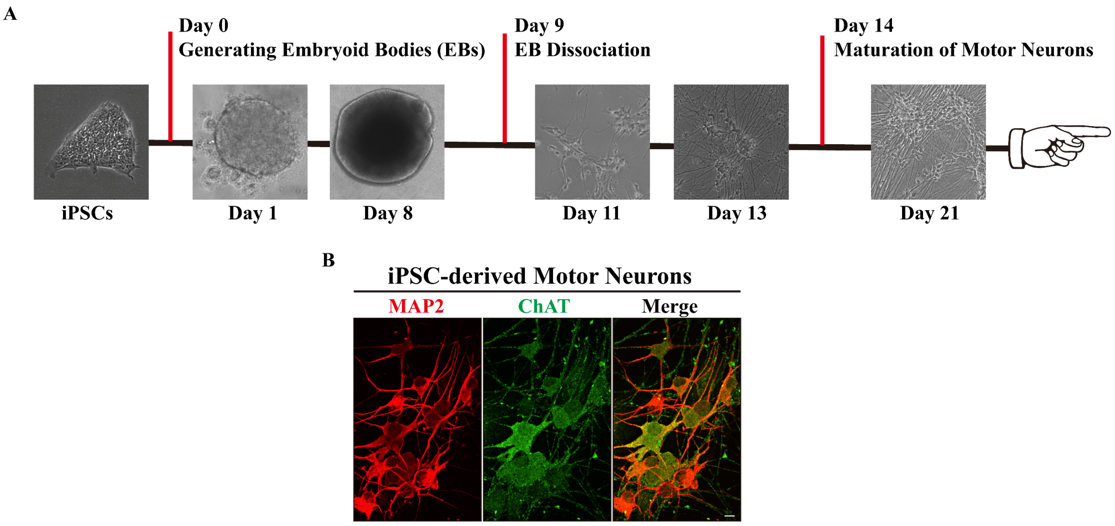

Figure S11. Related to Figure 7. (A) Timeline of generating motor neurons from iPSC. **(B)** Staining of neuronal marker MAP2 and motor neuronal marker ChAT after 27 days of differentiation and maturation. Scale bar, 10 μm.
